# Picosecond multilevel resistive switching in tantalum oxide thin films

**DOI:** 10.1038/s41598-020-73254-2

**Published:** 2020-10-02

**Authors:** Ulrich Böttger, Moritz von Witzleben, Viktor Havel, Karsten Fleck, Vikas Rana, Rainer Waser, Stephan Menzel

**Affiliations:** 1 Institut für Werkstoffe der Elektrotechnik (IWE 2) and JARA - Fundamentals for Future Information Technology, RWTH Aachen University, 52056 Aachen, Germany; 2 Peter Grünberg Institut PGI-10 and JARA - Fundamentals for Future Information Technology, Forschungszentrum Jülich, 52425 Jülich, Germany; 3 Peter Grünberg Institut PGI-7 and JARA - Fundamentals for Future Information Technology, Forschungszentrum Jülich, 52425 Jülich, Germany

**Keywords:** Information storage, Electrical and electronic engineering

## Abstract

The increasing demand for high-density data storage leads to an increasing interest in novel memory concepts with high scalability and the opportunity of storing multiple bits in one cell. A promising candidate is the redox-based resistive switch repositing the information in form of different resistance states. For reliable programming, the underlying physical parameters need to be understood. We reveal that the programmable resistance states are linked to internal series resistances and the fundamental nonlinear switching kinetics. The switching kinetics of $\hbox {Ta}_2 \hbox {O}_5$-based cells was investigated in a wide range over 15 orders of magnitude from 10$^5$ s to 250 ps. The capacitive charging time of our device limits the direct observation of the set time below 770 ps, however, we found indication for an intrinsic switching speed of 10 ps at a stimulus of 3 V. On all time scales, multi-bit data storage capabilities were demonstrated. The elucidated link between fundamental material properties and multi-bit data storage paves the way for designing resistive switches for memory and neuromorphic applications.

## Introduction

The class of redox-based resistive switching devices (ReRAM) based on the valence change mechanism (VCM) is a potential type for future non-volatile memory^1,2^, and computation-in-memory applications^3–7^. A typical VCM cell consists of a resistively switching oxide layer sandwiched between a high work function metal electrode such as Pt and a low work function metal, e. g. Ta. Among the numerous resistively switching oxides, $\hbox {Ta}_2 \hbox {O}_5$ is a promising material in terms of endurance^8^, scalability^9^, switching speed^10^, and multilevel switching capability^11^. Before the VCM cell can be switched repetitively between a high resistive state (HRS) and a low resistive state (LRS), an electroforming step is required. For this, a voltage is applied to the cell and the oxide thin film is locally reduced by extraction of oxygen resulting in a highly-conducting, oxygen-deficient filamentary region^1,12^.

The resistive switching effect has been attributed to a movement of mobile donors such as oxygen vacancies or cation interstitials, and a subsequent change in the filament composition leading to a valence change in the cation sublattice^1,13–15^. As the switching mechanism is dominated by the drift of ions, the switching operation is inherently bipolar. One voltage polarity is needed to set the cell from HRS to LRS, whereas the opposite voltage polarity is required to reset the device from LRS to HRS. Typically, an abrupt set transition is observed, whereas the reset transition is gradual^16–18^. The abrupt SET transition is a result of the local Joule heating in the filamentary region. As the device is initially in the HRS, only a small current flows through the device at the beginning of an electrical stimuli. This small current, however, increases the local temperature, which in turn increases the electrical conductivity. This results in a thermal runaway leading to an abrupt SET transition^19,20^. During the RESET Joule heating also occurs, and the oxygen vacancies drift away from the active electrode (high work function metal). The resulting depletion of oxygen vacancies at the active electrode decreases the electrical conductivity. Additionally, a diffusion current of oxygen vacancies toward the active electrode sets in. Both effects lead to the gradual RESET of VCM devices^17^.

The capability of multilevel operation, i. e. storing multiple bits per cell, enhances the storage density^21–23^. In that case, the programming process is stopped at a specific intermediate resistive state (IRS) and is controlled either by the applied voltage during the gradual reset of the cell^17,21,24^ or by a current compliance during the set operation^24,25^. For neuromorphic applications, the feature of multilevel switching is essential^26,27^.

In order to meet the needs for future non-volatile memories, the so-called voltage-time-dilemma has to be overcome^1^. This corresponds to an extremely nonlinear switching kinetics of the ReRAM cell characterized by a low-voltage read-out operation over a long period up to ten years and a fast write process in the nanosecond regime or below by applying a voltage that is about ten times higher than the read voltage. While several groups have studied the switching kinetics of ReRAMs in certain limited ranges as compiled in^28^, an investigation over the complete dynamic range has not been demonstrated yet. To cover the full time-domain, the measurements have to be extended to the sub-nanosecond regime, too. Resistive switching in the sub-nanosecond regime has been qualitatively demonstrated for VCM cells based on $\hbox {HfO}_2$^29^, $\hbox {Ta}_2 \hbox {O}_5$^10^, $\hbox {SiO}_2$^30^, and AlN^31^. The switching event, however, could not be resolved in these studies and the reproducibility of the switching on a single cell was rather low.

Here, we present a comprehensive study of the switching kinetics of $\hbox {Ta}_2 \hbox {O}_5$-based VCM cells from 250 ps to up to 10$^5$ s by the means of an optimized coplanar waveguide (CPW) device structure and the use of multiple measurement setups. This enables us to resolve the switching time over 15 orders of magnitude at the same VCM cell. The work is exclusively focused on the set process. The reset kinetic is also topic of the authors’ current work and will be published separately.

Furthermore, we demonstrate highly reproducible multilevel programming performed by varying the amplitude and length of the pulse. The data analysis reveals that the programmed LRS is linked to the inherent nonlinear VCM switching kinetics and an internal series resistance. Based on this finding, we discuss design rules for optimizing the multilevel programming capability of VCM cells integrated with a passive selector.

## Results

### Effects of series resistances

**Figure 1:**
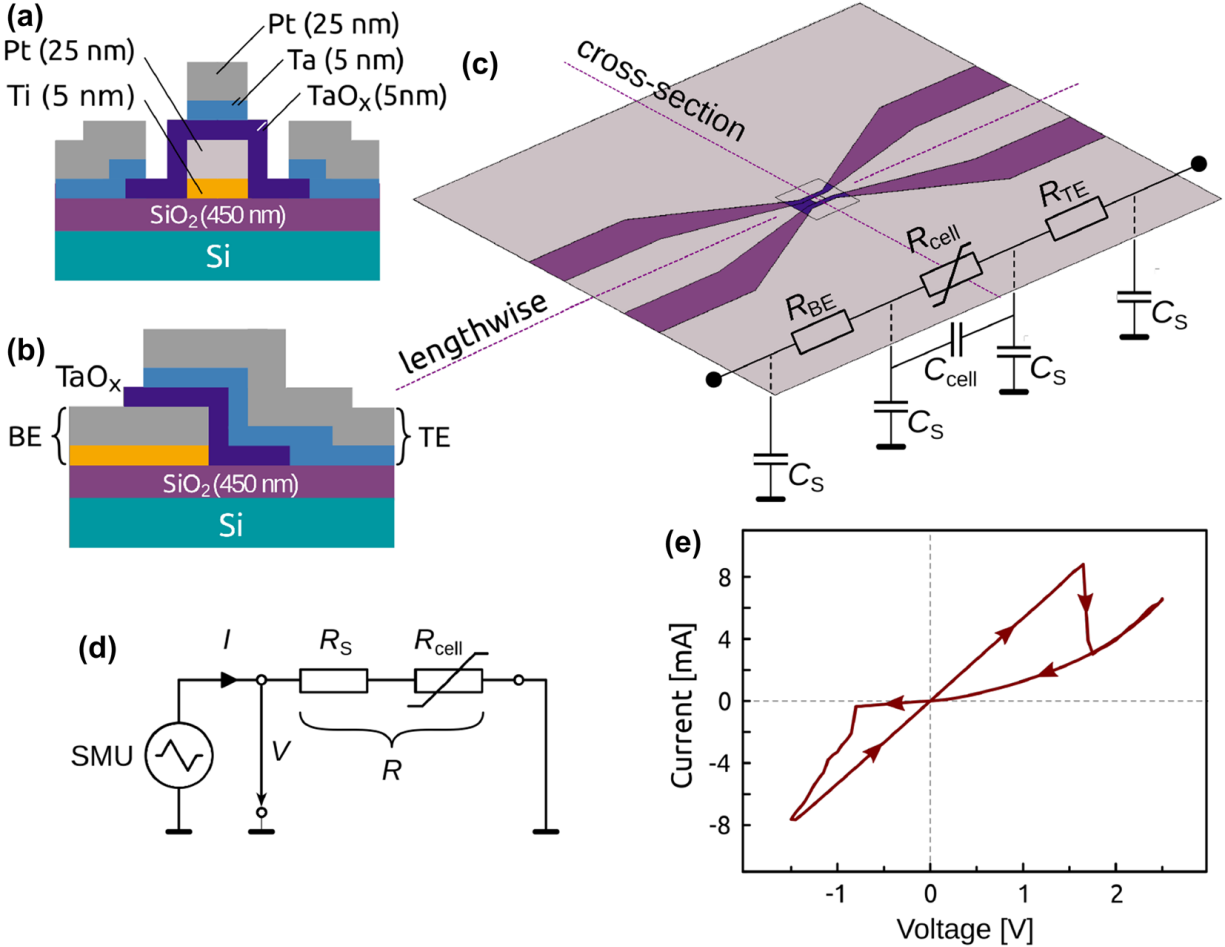
Schematic overview of the $\hbox {Ta}_2 \hbox {O}_5$ ReRAM cell showing (**a**) the cross-section, (**b**) the lengthwise arrangement, and (**c**) the integrated structure with overlapping electrodes including the corresponding *RC* network. (**d**) Equivalent circuit diagram with the cell resistance ${R}_{\mathrm{cell}}$ and the serial resistance ${R}_{\mathrm{S}} = R_{\mathrm{BE}} + {R}_{\mathrm{TE}}$ for quasi-static characterization, and (e) *I*(*V*)-sweep characteristic of the CPW device. A gradual set is observed between $-0.9\,$ V$<V< -1.6$ V, an abrupt reset appears at $V\approx +1.6$ V.

The schemes in Fig. 1a–d show the investigated, tapered CPW structures with $\hbox {Ta}_2 \hbox {O}_5$ ReRAM cells optimized in terms of high frequency impedance matching. The tapered design constantly maintains the impedance ($50\,\Omega$) of the cables and probes along the lines when the dimensions of the CPW, needed for contacting by probe tips, are reduced to a smaller area sizes of the cell. This approach inhibits reflections at the contacts and is used in other studies^10,30,31^. Cells with area sizes of $A_1 = 15 \times 20\, \upmu$m$^2$ and $A_2 = 20 \times 30\, \upmu$m$^2$ were measured. The fabrication process of the layer stack is identical with that of our previous work^32^ and leads to amorphous $\hbox {Ta}_2 \hbox {O}_5$ films (cf. Sample preparation). The equivalent circuit of the entire device includes the variable resistance of the $\hbox {Ta}_2 \hbox {O}_5$ layer ${R}_{\mathrm{cell}}$ and the series resistance ${R}_{\mathrm{S}}$ combining the contributions of electrodes, electrical lines, and the contacts of the bottom and top electrode path. By electrical characterization, only the total device resistance *R* of the entire device is measurable: $R= {R}_{\mathrm{S}} + {R}_{\mathrm{cell}}$ (Fig. 1d).

The existence of ${R}_{\mathrm{S}}$ leads to the fact that in a quasi-static *I*(*V*)-curve the transition changes its abrupt characteristic. The switching starts at a specific negative voltage. When the cell resistance decreases during set operation, it approaches the range of the series resistance. The applied voltage $V = V_{\mathrm{S}}+V_{\mathrm{cell}}$ will be redistributed between $R_{\mathrm{cell}}$ and $R_{\mathrm{S}}$. In consequence, the cell voltage $V_{\mathrm{cell}}$, and therefore, the driving force for resistance reduction decreases until the process finally grinds to a halt in the timeframe of the experiment at a defined voltage $V_{\mathrm{min}}$^33–35^. So, the characteristic abrupt set transition of VCM devices appears as a gradual transition. This behavior is also observed for the CPW devices under test (DUT), see Fig. 1e. The gradual set transition does not necessarily have to originate from the internal series resistances, it may also be caused by external series resistances^33,36,37^. It is most essential that ${R}_{\mathrm{S}}$ is independent of the applied voltage.

In a similar manner the reset operation of the ReRAM cell is modified by the series resistance, see Fig. 1e. In case of the application of a positive voltage to the device in the LRS, the series resistance is dominant and the applied voltage mainly drops over ${R}_{\mathrm{S}}$. As soon as the cell resistance increases during the reset operation, the ratio of the voltage divider changes and causes a positive feedback, i. e. the cell voltage increases. The reset process speeds up and the transition becomes abrupt. The series resistance, hence, masks the intrinsic abrupt set and gradual reset behavior of the VCM cell and turns it into a gradual set and an abrupt reset process^36^.

### Ultra-fast multilevel switching

**Figure 2:**
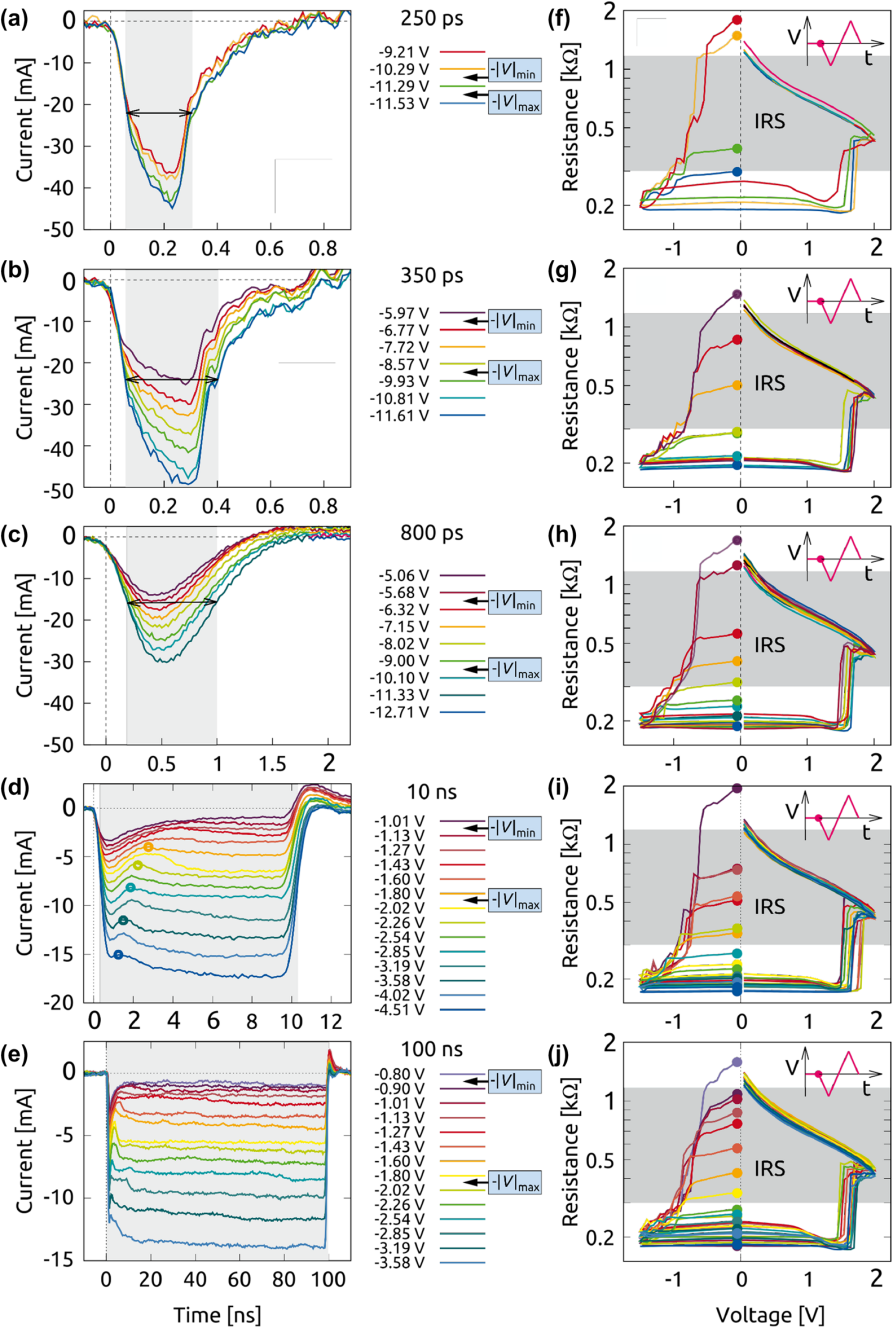
(**a**–**e**) Transient pulse measurements of a $\hbox {Ta}_2 \hbox {O}_5$ CPW device stack with $A_2$ in the picosecond and nanosecond time range for a series of set pulses with variable pulse amplitude $|V_p|$ and (**f**–**j**) the corresponding subsequent *R*(*V*)-sweep measurements on the same device after applying the write (set) pulses. The *R*(*V*) curves start at the dot at $-0.05$ V either in the HRS ($R > 1.2$ k$\Omega$) or IRS (grayed-out area) or LRS ($R < 300$ $\Omega$) and end in the HRS for all write pulses. The multilevel operation is obvious.

A series of ultra-short set pulses with lengths between 250 ps and 100 ns and amplitudes up to 12.7 V were applied to the CPW devices in the HRS. The transients in Fig. 2a–e show the corresponding waveforms of the current through a cell with an area size $A_2$. For the 10 ns and 100 ns pulses, the switching events for each amplitude are clearly identified and are exemplarily illustrated by the marked inflection points in Fig. 2d. The general trend shows faster switching for increasing pulse amplitudes. No inflection point, i. e. no switching event, is observed as long as the absolute value $|V_p|$ is below a minimum voltage $|V|_{\mathrm{min}}$. Overshoots by charging and discharging the cell capacitance predominantly determine the transient currents over the full time range.

The response on picosecond pulses is without any signature of a possibly happened switching event (Fig. 2a–c). This originates from the large device capacitance, whose charging affects the signal waveform and makes the signal changes of higher bandwidth undetectable. A similar behavior was also found by Torrezan et al.^10^. Nevertheless, even in case of 250 ps pulses a “complete” switching from the HRS into the LRS was clearly demonstrated.

The verification of the resistance reduction after pulsing was carried out by subsequent *I*(*V*)-sweeps with a linear rate of 0.1 V/s, which start with the same negative polarity as the set pulse. The resulting *R*(*V*) behavior is illustrated in Fig. 2f–j. For low pulse amplitudes $|V_{p}| \le |V|_{\mathrm{min}}$, the *I*(*V*)-sweep of the ReRAM cell starts in the HRS ($R > 1.2$ k$\Omega$) because the stimulus of the prior fast set pulse was not sufficiently strong enough to induce the switching process. In the range $|V|_{\mathrm{min}}< |V_p| < |V|_{\mathrm{max}}$ the cell is switched partially to an intermediate state, whose resistances monotonically decrease with increasing pulse amplitudes $|V_p|$. The cell is switched fully to the LRS defined here as $R < 300$ $\Omega$, since $|V_p| \ge |V|_{\mathrm{max}}$. Further voltage enhancement will result in no or little resistance decrease. The voltage limits for the different pulse lengths can be estimated from Fig. 2.

The programming of different resistance states by amplitude modulation was so far only observed for pulse lengths of 100 ns or longer, e. g.^38^. Here, we could demonstrate for the first time the multilevel set capability even with picosecond pulses. This behavior was confirmed for different cells at various sizes. The investigated tantalum oxide ReRAM cells are prepared under identical processing conditions as the samples used in^11^ by our group where their (long-pulse) multi-bit feasibility as well as their retention behavior up to 10$^4$ s was already shown.

### Extended time domain measurements

**Figure 3:**
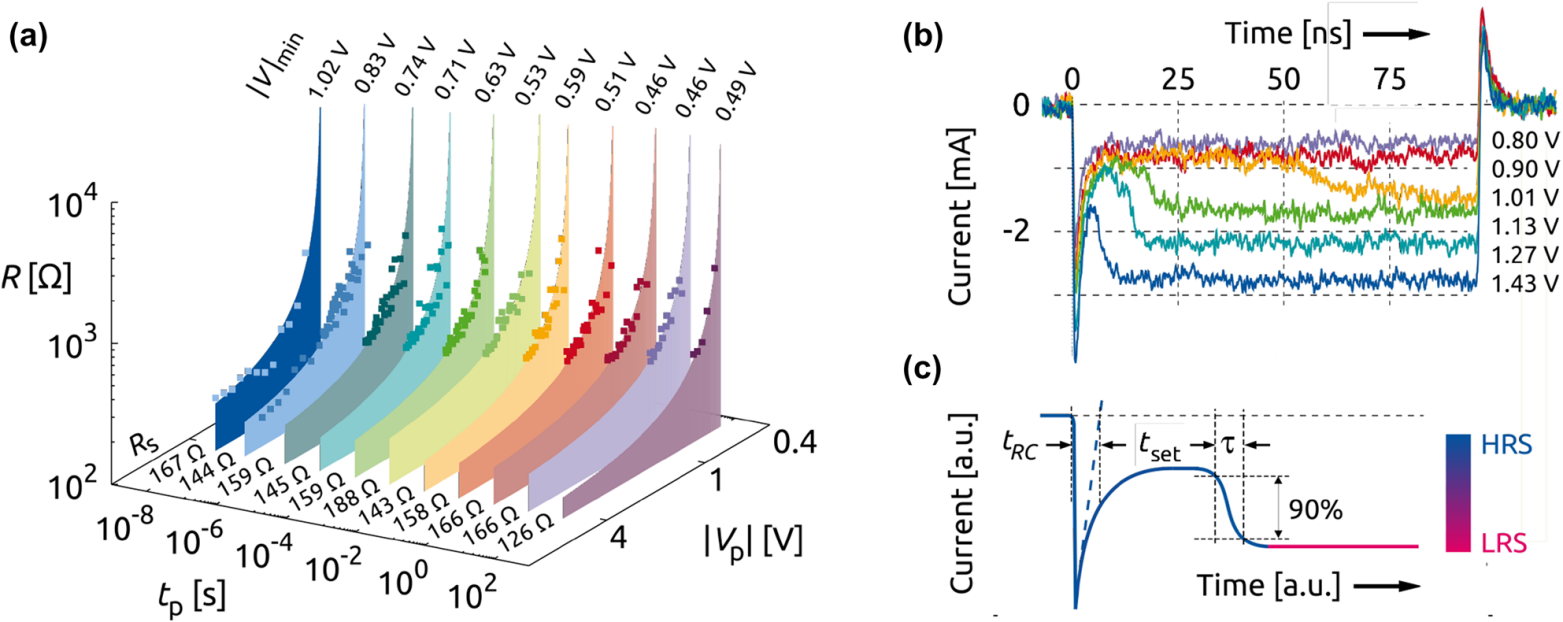
(**a**) 3D point plot of the measured data ($R,V_p$) of a $\hbox {Ta}_2 \hbox {O}_5$ CPW cell with an area size $A_1$ for a pulse width $t_p$, and fitted behavior of the programmed resistance state *R* using the fit parameters $|V|_{\mathrm{min}}$ and ${R}_{\mathrm{S}}$ pursuant to Eq. (2). (**b**) Corresponding current response on 100 ns pulses with different amplitudes and (**c**) Illustration of charging time $t_{RC}$, set time $t_{\text {set}}$, and transition time $\tau$ for an idealized switching behaviour from the HRS to the LRS.

The expansion of the investigation to pulse lengths up to 10$^5$ s reveals that programming the LRS or one of the IRS by controlling the pulse amplitude is possible on all time scales. The smaller cell with $A_1$ was measured, which has only a capacitance of $C_{\mathrm{cell}} = 4.6$ pF. In Fig. 3a the programmed resistance *R* is plotted versus the absolute value of the applied pulse voltage $|V_p|$ for different pulse widths $t_p$. Two trends can be observed: (i) *R* appears to be inversely proportional to $|V_p|$ for all $t_p$, and (ii) the programmed resistance becomes lower for longer pulse widths.

As already mentioned, the series resistance reduces the voltage drop over the memory cell during set operation and leads to a certain minimum voltage $|V|_{\mathrm{min}}$, at which the driving force for further resistance reduction of $R_{\mathrm{cell}}$ becomes practically zero. The fact that *R* depends on $t_p$ implies that $|V|_{\mathrm{min}}$ depends on $t_p$, too. Thus, $|V|_{\mathrm{min}}$ should be linked somehow to the switching kinetics of the device. As long as the voltage does not exceed $|V|_{\mathrm{min}}$, the cell stays in the HRS and the maximum current during set operation for a given $t_p$ can be described by Kirchhoff‘s current law:

1 $$\begin{aligned} |I|_{\mathrm{max}} = \displaystyle \frac{|V(t_{p})|_{\mathrm{min}}}{{R}_{\mathrm{cell}}}= \displaystyle \frac{|V_p|}{R}= \displaystyle \frac{|V_p|-|V(t_{p})|_{\mathrm{min}}}{{R}_{\mathrm{S}}}. \end{aligned}$$

Reformulating Eq. (1) provides an expression for the programmed resistance state

2 $$\begin{aligned} R = \displaystyle \frac{|V_p|}{|V_p|-|V(t_{p})|_{\mathrm{min}}} \, {{R}_{\mathrm{S}}}= \displaystyle \frac{{R}_{\mathrm{S}}}{1 - |V(t_{p})|_{\mathrm{min}}/|V_p|}, \end{aligned}$$

which is a function of the pulse voltage, the series resistance and the minimum voltage. Corresponding to a given pulse length $t_p$, each set of data is fitted to the curve $R(V_p)$ applying the fit parameter $|V(t_{p})|_{\mathrm{min}}$ and ${{R}_{\mathrm{S}}}$ by minimizing the least mean square error. As depicted in Fig. 3a, the fitted series resistances are (averaged for pulse lengths $10^{-8}$ s $\le t_{p} \le 10^{1}$ s) close to the value ${R}_{\mathrm{S}} \approx 160$ $\Omega$. The resulting $R(V_p)$-behavior is represented by the top edge of the colored areas in Fig. 3a and match the experimental data well. In addition, it should be noticed that the estimated values of the minimum voltage for 10 ns and 100 ns pulses of Fig. 2 are in good agreement with the fit parameter $| V(t_{p})|_{\mathrm{min}}$ of Fig. 3a.

A typical current response of the $\hbox {Ta}_2 \hbox {O}_5$ CPW cell with $A_1$ during a 100 ns pulse is shown in Fig. 3b. After the occurrence of a capacitive current during the characteristic charging time $t_{\mathrm{RC}}$, the current remains initially constant in the HRS before it increases in a transition time $\tau$ as illustrated in Fig. 3c. Following the definition from previous publications^39^, the switching time $t_{\mathrm{set}}$ is given as the interval between the moment the cell is charged up to 63 % and the onset of the current rise. For moderate pulse voltages with lengths larger than 100 ns, $\tau$ is in the order of nanoseconds or hundreds of them depending on the applied voltage^16,40^. The transition time describes the current runaway, i. e. the resistance reduction, in the moment of switching and is defined as the period from the current rise to the reaching of the 90 % level of the final value, which may be the LRS or one of the IRS^41,42^. As already mentioned, in case of ultra-short pulses, a clear identification of the characteristic times is no longer possible. The transition starts before the cell is fully charged or even during the rising edge of the voltage pulse.

## Discussion

**Figure 4:**
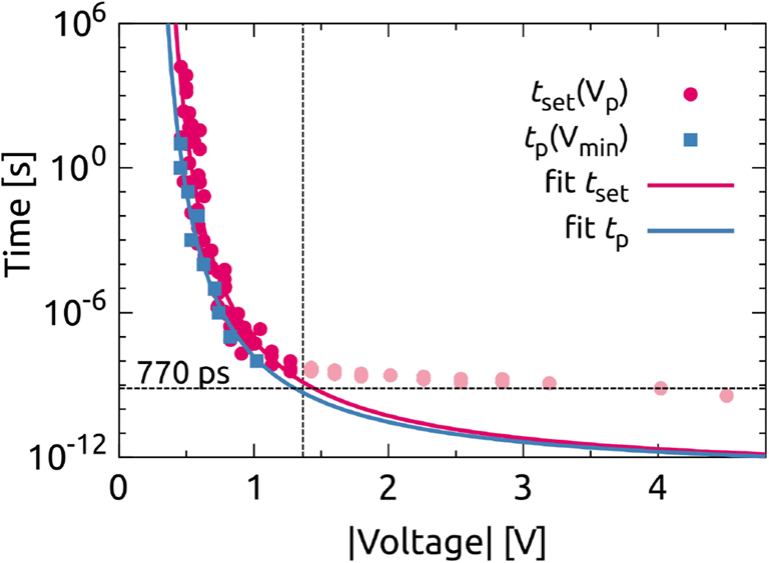
Non-linear switching kinetics of a $\hbox {Ta}_2 \hbox {O}_5$ CPW cell with $A_1$ by the means of a $t_{\mathrm{set}}(V_p)$ plot over approximately 15 decades at the time scale (dark red and light red circles), and corresponding fit to Eq. (3) for $V<1.4$ V (red line). Furthermore, $t_{p}(V_{\mathrm{min}})$ is plotted for each $t_p$ of Fig. 3a (blue squares) and fitted according to Eq. (3) (blue line). The measurement limit is given by the *RC* time (770 ps) of the device.

By the combination of the results of different time regimes, the strong dependence of the set switching time on the pulse amplitude can be illustrated over 15 orders of magnitude (Fig. 4). Each red colored data point represents a resistive switching event from the HRS to a state of higher conductance, which may be either the LRS or one of the IRS. To the best of the authors’ knowledge it is the first time that such a high dynamic range of the switching kinetics including the picosecond regime is presented.

In the voltage range $|V| < 1.4$ V (Fig. 4), the experimental data show a very strong nonlinearity following the empirical relation

3 $$\begin{aligned} t_{\mathrm{set}} = t_0 \displaystyle \exp \left( \frac{\kappa }{|V_p|-V_0}\right) \end{aligned}$$

with the fit parameters $\kappa =11.2$ V and $V_0=0.162$ V. The parameter $t_0 = 1.19\times 10^{-13}$ s is equivalent to a wavenumber ${\tilde{\nu }}=280\,\mathrm{cm}^{-1}$ for amorphous $\hbox {Ta}_2 \hbox {O}_5$, which was found for the deformation modes of the Ta−O−Ta and Ta$\equiv$O bonds by infrared absorption spectroscopy^43^. Based on the suggestion that the electric-thermally activated migration of oxygen vacancies in $\hbox {Ta}_2 \hbox {O}_5$ thin films is the responsible switching mechanism, the phonon vibrations represent the lower limit of the switching time for high voltages $V_p\rightarrow \infty$. The same behavior was theoretically found in our previous study in which the oxygen vacancy movement was described by the Mott–Gurney Law^44^.

In the voltage range $|V| > 1.4$ V, which corresponds to shorter pulses, the measured behavior in Fig. 4 deviates from the expected one and the course of data points flattens towards slower switching times with increasing pulse amplitude. This is due to the fact of the non-neglectable *RC* time of the device. The equivalent circuit shown in Fig. 1c–d comprises the series resistance $R_{\mathrm{S}}$, the capacitances between the CPW electrodes and the ground planes $C_{\mathrm{S}}$, the capacitance of the ReRAM cell $C_{\mathrm{cell}}$ and the time-dependent cell resistance $R_{\mathrm{cell}}(t)$. The time-invariant capacitances are determined with impedance measurements at 1 MHz for a cell area $A_1$ and amount to $C_{\mathrm{S}} = 10.6$ pF and to $C_{\mathrm{cell}} =4.6$ pF. Using the fit parameter $R_{\mathrm{S}} = 167$ $\Omega$ for the series of 10 ns pulses of Fig. 3a results in $t_{RC} = 770$ ps.

The switching time is, consequently, not limited at 250 ps by internal physical processes, such as the migration of oxygen vacancies^19^, but by the capacitive charging of the cell. It was already shown in^10^ that faster SET times down to 105 ps are possible in $\hbox {Ta}_2 \hbox {O}_5$ devices, which coincides also with the limit of their setup. Based on these facts, we believe that faster SET times down to tens of picoseconds are realizable in ReRAM devices. Thus, the measured data pairs ($|V_p|,t_{\mathrm{set}}$) represent an upper limit of the set time at a given pulse height. An improvement of the measurement accuracy could be possible by the means of *RC* reduction by decreasing the cell capacitance area. However, such an approach may run into a more pronounced impedance mismatch causing a stronger damping of the transmitted signal and a worse temporal resolution. In an ideal case ($t_{RC} \rightarrow 0$), the extrapolated behavior in Fig. 4 indicates an internal switching speed of about 10 ps for $|V| \approx 3$ V.

The blue colored data pairs $t_{p}(V_{\mathrm{min}})$ in Fig. 4 illustrate the relation between pulse width and minimum voltage taken from Fig. 3a. In fact, these data points behave similarly to $t_{\mathrm{set}}(V_p)$ and can be fitted in a similar way via Eq. (3) with the parameter set $t_0 = 1.10\times 10^{-13}$ s, $\kappa =10.3$ V, and $V_0=0.124$ V. The resulting curve lies slightly below the switching kinetics data. For a given $V_{\mathrm{min}}$, the corresponding $t_{p}$ represents the moment, at which the switching does not occur anymore. Otherwise, for a given $t_{p}$, the corresponding $V_{\mathrm{min}}$ marks the voltage at which the transition halts. This analysis reveals the link between programmable resistance states and the intrinsic switching kinetics of the ReRAM cell.

According to Eq. (2) and assuming an invariant internal series resistance, the programmed resistance at a specific pulse width is determined by the applied voltage and the minimum voltage. Pulse width and minimum voltage, however, are not independent of each other due to the switching kinetics. If the kinetics is strongly non-linear as it is indicated by the steep slope in the log(*t*)-*V*-diagram of Fig. 4 for $|V|_{\mathrm{min}} < 1$ V and $t_{p}>100$ ns, the minimum voltage is almost constant for all $t_{p}$ and the programmed resistance predominantly depends on the pulse voltage amplitude. For a weak non-linearity, i. e. a flat slope $d\log (t)/dV$, an additional dependence of *R* on the time scale is present because of the sensitivity of $|V|_{\mathrm{min}}$ to $t_{p}$. From this point of view, a highly nonlinear switching kinetics will be beneficial in terms of variability, which is permanently of major interest for resistive switching cells^45^.

As pointed out in^36,37^, the voltage divider effect caused by an external resistance improves the variability and the device endurance. For multilevel programming, however, a slight voltage variation close to $|V|_{\mathrm{min}}$ could evoke a larger (not acceptable) resistance variability. Thus, the pulse amplitude $|V_p|$ should be sufficient higher than $|V|_{\mathrm{min}}$. As the series resistance is linear, the resistances programmed with different voltages lie close to each other. In a big array these different resistance states might be indistinguishable considering cell-to-cell variability^46^. A potential strategy to overcome this problem is the use of a nonlinear series resistance.

Without additional elements, the multilevel programming of our devices is only feasible for the set operation. As already explained above, the reset is an abrupt transition from the LRS into the HRS due to the voltage divider effect. For neuromorphic applications, however, it is desirable to program different resistances during the set as well as during the reset operation. An suitable approach is the reduction of the voltage divider effect to emphasize the intrinsic gradual reset transition, e. g. by introducing a selector element with an asymmetric *I*-*V* characteristics^47,48^. For the set mode, this selector should limit the current and define the programmed resistance. For the reset mode, the selector should be highly conducting, so that the applied voltage would drop completely over the actual resistively switching element and the intrinsic gradual reset transition appears. In this way, multilevel programming capabilities could be achieved for both voltage polarities.

In this work, the multilevel resistive switching of $\hbox {Ta}_2 \hbox {O}_5$ cells at pulse lengths down to 250 ps was presented. For nanosecond pulses the monitoring of transient currents enables us to resolve the set switching event, and to find a clear dependence between the applied voltage and the resulting switching time. In combination with long pulse experiments, the non-linearity of the switching kinetics over 15 orders of magnitudes was demonstrated. For pulse lengths longer several ns the migration of oxygen vacancies is the limiting parameter, for shorter pulses the *RC* time of the set-up restricts the switching speed. Nevertheless, the over-all behavior implies the overcome of the voltage time dilemma, which is essential for the use of any resistive two-terminal devices. The multilevel capability together with the high intrinsic switching time of a single bit, which was estimated with 10 ps at 3 V without any parasitic effects, provides the option to store multiple bits per cell in a time regime down to 100 ps, which is significantly faster than writing times of state-of-the-art memory devices.

## Methods

**Sample preparation.** ReRAM devices were fabricated—based on the work in^32^—by integrating a 5 nm thin $\hbox {Ta}_2 \hbox {O}_5$ film into a tapered 50 $\Omega$ CPW structure designed for impedance matching of the high frequency coaxial coplanar probes (150 $\upmu$m pitch). High-resistivity substrates of silicon (CrysTec GmbH, 4“ $\left<100\right>$ wafers, $\rho>$ 10 k$\Omega$ cm) with 450 nm thermally grown $\hbox {SiO}_2$ were used. The bottom electrodes consisting of 5 nm Ti (adhesion layer) and 25 nm Pt were realized by DC-sputtering and patterned by ion beam etching. The deposition of the $\hbox {Ta}_2 \hbox {O}_5$ was carried out via RF-sputtering from a metallic target with 2% oxygen and subsequent structuring by reactive ion beam etching process. The top electrodes metals (5 nm Ta and 25 nm Pt) were fabricated by e-beam evaporation and lift-off lithography. All deposition processes are performed at room temperature. Devices with effective cell areas $A_1 = 300\,\upmu$m$^2$ and $A_2 = 600\,\upmu$m$^2$ corresponding to the overlap of inner signal CPW line were processed on single wafers. Impedance measurements (1 MHz) of cell areas $A_1$ result in $C_{\text {cell}} = 4.6$ pF which is linked to a dielectric constant $\varepsilon _{\text {r}}\approx 8$ which is far away from values $\varepsilon _{\text {r}} > 30$ for different polymorphs of crystalline $\hbox {Ta}_2 \hbox {O}_5$^49^. Therefore, it is concluded that the deposited films are amorphous. All samples were initially electroformed in the HRS by a triangular positive voltage sweep using Keithley 2634B Source-Meter with an amplitude of $+4$ V and 100  $\upmu$A current compliance.

### Transient current response

The range from $10^{-7}$ to $10^2$ s was characterized by pulse measurements performed with a Keithley 4200-SCS semiconductor characterization system with a 4225-PMU ultra-fast I/V modules and two 4225-RPM remote amplifiers. The transients were analyzed in terms of $t_{\mathrm{set}}$ as depicted in Fig. 3c, see also^42,50^. The pulse amplitude was gradually reduced from − 1 V down to − 0.3 V and the pulse length proportionally varied from 100 ns to 100 s. For the measurements between 1 s and 10$^5$ s a Keithley 2636A Source-Meter was used. DC voltage was applied to the DUT (device under test) and the current was concurrently monitored. The voltage was varied from − 0.6 V to − 0.2 V in steps of 20 mV. After the detection of a significant current increase, 200 data points were subsequently recorded until the measurement was stopped. $t_{\mathrm{set}}$ was determined by use of the same algorithm as on the Keithley 4200-SCS setup.

### Ultra-fast pulse measurements

Pulse generation in the nanosecond and picosecond regime applies different setups mainly based on the suggestions of Torrezan et al.^10^: (i) a Picosecond Pulse Labs 2600C with a variable amplitude of $-45-+50$ V at 0 dB attenuation generates pulses with widths in the range from 0.8 ns to 100 ns. The pulse amplitude can be attenuated in 1 dB steps from 0 to 70 dB. The output signal is divided by a power splitter into two identical pulses. The first part delivers the reference signal and the second pulse is guided through the DUT. (ii) pulses down to 250 ps are generated by a Picosecond Pulse Labs 12050 pattern generator producing a continuous pattern of pulses with a width $t_p=78$ ps, a height up to 750 mV, and a repetition rate of $\tau _r=41$ $\upmu$s. It is combined with an appropriate timed RF switch for coupling of single pulses to the DUT. In order to provide a sufficient high set signal a PSPL 5868 RF-amplifier with 12 V output at high impedance load is needed. The RF switch and the RF amplifier as well as the device capacitance limit the bandwidth of the system. Therefore, the minimal 78 ps pulse width is widened to 100 ps. The output signal is fed directly to the DUT (without division) since the signal reduction by the power splitter would be so strong that the required switching voltage would not be reached. Due to the capacitances of the DUT, the measured current response broadens to 250 ps. The resistance of the device is in the HRS much greater than 50 $\Omega$. Therefore the applied voltage of the SET pulse can be considered as double of the voltage assumed for an ideal 50 $\Omega$ termination. In both cases the signal from the DUT is captured with an oscilloscope Tektronix DPO 73304D, 33 GHz, 100 GS/s, real time oscilloscope with 50 $\Omega$ input terminations. The presented transients show the current through the DUT, proportional to the voltage over the scope input. Subsequent *I*(*V*) measurements by a Keithley 2634B were used to determine the resistance state after pulsing.
